# Altered life history strategies protect malaria parasites against drugs

**DOI:** 10.1111/eva.12516

**Published:** 2017-11-06

**Authors:** Philip L. G. Birget, Megan A. Greischar, Sarah E. Reece, Nicole Mideo

**Affiliations:** ^1^ Institutes of Evolutionary Biology, Immunology and Infection Research University of Edinburgh Edinburgh UK; ^2^ Department of Ecology & Evolutionary Biology University of Toronto Toronto ON Canada

**Keywords:** gametocytes, life history evolution, nonclassical drug resistance, *Plasmodium*, pyrimethamine, transmission investment

## Abstract

Drug resistance has been reported against all antimalarial drugs, and while parasites can evolve classical resistance mechanisms (e.g., efflux pumps), it is also possible that changes in life history traits could help parasites evade the effects of treatment. The life history of malaria parasites is governed by an intrinsic resource allocation problem: specialized stages are required for transmission, but producing these stages comes at the cost of producing fewer of the forms required for within‐host survival. Drug treatment, by design, alters the probability of within‐host survival, and so should alter the costs and benefits of investing in transmission. Here, we use a within‐host model of malaria infection to predict optimal patterns of investment in transmission in the face of different drug treatment regimes and determine the extent to which alternative patterns of investment can buffer the fitness loss due to drugs. We show that over a range of drug doses, parasites are predicted to adopt “reproductive restraint” (investing more in asexual replication and less in transmission) to maximize fitness. By doing so, parasites recoup some of the fitness loss imposed by drugs, though as may be expected, increasing dose reduces the extent to which altered patterns of transmission investment can benefit parasites. We show that adaptation to drug‐treated infections could result in more virulent infections in untreated hosts. This work emphasizes that in addition to classical resistance mechanisms, drug treatment generates selection for altered parasite life history. Understanding how any shifts in life history will alter the efficacy of drugs, as well as any limitations on such shifts, is important for evaluating and predicting the consequences of drug treatment.

## 1 INTRODUCTION

Malaria parasites (*Plasmodium* spp.) remain one of the most severe and common causes of human disease (White, Pukrittayakamee, et al., 2014). Although interventions against malaria parasites have seen significant successes over the last 30 years (WHO 2015a), resistance has evolved to every antimalarial drug in widespread use (Hyde, 2005; White, 2004; WHO 2015a). In many cases, this resistance has been attributed to “classical” resistance mechanisms (sensu Schneider et al., 2012), including target site mutations or detoxification mechanisms (Hyde, 2002, 2005). However, changes in parasite behaviour, metabolism or life history, that is, “nonclassical” resistance mechanisms (Schneider et al., 2012), offer additional threats to drug efficacy.

One potential mechanism for nonclassical resistance is evolving traits that give rise to higher within‐host parasite densities; this may offer protection against drugs by increasing the likelihood that some (genetically identical) parasites survive treatment (White, 1998). Experimental rodent malaria infections confirm that more virulent parasite strains, with faster within‐host replication, survive better in drug‐treated hosts (Schneider, Chan, Reece, & Read, 2008; Schneider et al., 2012). But within‐host densities are at least in part governed by a resource allocation trade‐off in malaria and other sexually reproducing parasites: achieving higher within‐host densities comes at the cost of producing fewer specialized sexual stages (gametocytes) that are required for transmission (Carter et al., 2013; Pollitt et al., 2011), as a parasite in a given infected host cell can follow only one of the two developmental routes. Transmission investment—by convention referred to as the conversion rate—varies plastically within artificial culture, increasing as conditions become more crowded (Bruce, Alano, Duthie, & Carter, 1990). While conversion rate can change plastically in response to changing environmental conditions, data suggest that there is parasite genetic variation for patterns of conversion (Pollitt et al., 2011; Birget, Repton, O’Donnel, Schneider, & Reece, 2017) and that this variation can be selected upon (reviewed in Bousema & Drakeley, 2011). It is well known, for example, that serial passage and culture experiments, which by their nature select for faster within‐host replication, result in reduced transmission investment (Dearsly, Sinden, & Self, 1990; Sinha et al., 2014; reviewed in Carter et al., 2013). Similarly, artificial selection for attenuation in a related parasite, *Eimeria*, resulted in indirect selection for earlier investment in transmission, which translated into a substantial reduction in total transmission potential (McDonald & Shirley, 2009). Therefore, conversion rates represent an evolvable parasite trait essential to transmission, and the challenge is to explore if and how drug treatment might alter parasite strategies.

Malaria parasites appear to vary transmission investment in ways thought to be adaptive (Carter et al., 2013), and theory is an essential check on intuition regarding the fitness consequences of different strategies (Greischar, Reece, & Mideo, 2016). Models have shown that reducing transmission investment—though it might appear maladaptive (Taylor & Read, 1997)—can dramatically enhance parasite fitness by increasing the parasite numbers available to produce gametocytes later on and by improving persistence in the face of immunity and competing strains (Greischar, Mideo, Read, & Bjornstad, 2016a; Greischar, Read, & Bjørnstad, 2016; Koella & Antia, 1995; McKenzie & Bossert, 1998; Mideo & Day, 2008). It remains challenging to show experimentally that these predicted patterns are adaptive, actually improving parasite fitness in the face of environmental change, as techniques for forcing parasites to make alternative life history decisions are currently not available. However, the development of improved statistical methods now allows more accurate estimates of conversion rates in vivo (Greischar, Mideo, Read, & Bjornstad, 2016b), and theory is urgently needed to form clear expectations to compare with natural patterns. In contrast, conversion rates are comparatively easy to integrate into mathematical models by simply varying allocation to asexual growth and gametocyte production. Mathematical models demonstrate that changing allocation patterns can have significant impacts on parasite fitness (i.e., transmission potential) and can predict the optimal pattern in different environments (Greischar et al., 2014; Greischar et al., 2016a; Koella & Antia, 1995; McKenzie & Bossert, 1998; Mideo & Day, 2008). Understanding how selection imposed by drugs may alter transmission investment is critical, as any changes will have both clinical and epidemiological consequences.

Here, we predict the resource allocation patterns of malaria parasites that maximize fitness in drug‐treated hosts. We extend a previously published mechanistic model of within‐host malaria infection (Greischar et al., 2014; Greischar et al., 2016a) and use numerical optimization techniques to determine optimal conversion rates, that is, the proportion of infected host cells that produce transmission stages. Into this framework, we incorporate a simple model of drug action that was parameterized for the treatment of experimental rodent malaria infections with the antimalarial drug pyrimethamine (Huijben et al., 2013). By holding constant the duration and timing of drug treatment, but varying dose, this heuristic model allows us to explore the predicted impact of treatment of variable efficacy—from small to large reductions in parasite load—on parasite life history evolution. We explore optimal investment in transmission stages, first, by assuming parasites are constrained to a constant conversion rate throughout infections and, second, by permitting parasites to employ time‐varying conversion rates. Finally, we quantify the extent to which altering life history according to these optimal patterns can buffer against the effects of drugs and we evaluate the consequences for host health and onward transmission.

## 2 METHODS

### 2.1 The model

Following Greischar et al. (2014, 2016a), we use delay‐differential equations to model the within‐host dynamics of a malaria infection, which tracks uninfected red blood cells (*R*), infected red blood cells (*I*), extracellular malaria parasites (merozoites, *M*) and gametocytes (*G*). The change in density of uninfected red blood cells (RBCs) over time, *t*, is given by

(1) $$\frac{dR}{dt}=\lambda \left(\frac{1-R(t)}{K}\right)-\mu R\left(t\right)-pR\left(t\right)M\left(t\right).$$

The first term represents production of new RBCs by the host. Erythropoiesis is assumed to be a logistic function of current RBC density, where λ is the maximum realized rate of replenishing depleted RBCs and *K* determines the homeostatic equilibrium density. We assume that only uninfected RBCs count towards the homeostatic equilibrium because malaria parasites consume large amounts of haemoglobin during their development (e.g., Lew, 2003) and compromise the ability of infected RBCs to carry oxygen (Schmidt, Correa, Boning, Ehrich, & Kruger, 1994). We have found that including infected RBCs in this term makes little qualitative difference. In the absence of infection, RBC production balances natural death (which occurs at a rate, μ), so $K=\frac{\lambda R^{*}}{\lambda -\mu R^{*}}$, where R* represents the RBC density at homeostatic equilibrium. The final term represents a mass action infection process, and *p* is the rate at which merozoites invade RBCs upon contact.

The dynamics of infected RBCs are given by

(2) $$\frac{dI}{dt}=pR\left(t\right)M\left(t\right)-\mu I-pR\left(t-\alpha \right)M\left(t-\alpha \right)S$$

where *S* indicates the proportion of infected RBCs surviving development, equal to $e^{-\mu \alpha }$ when t>α and in the absence of drugs. An infected cell is generated when a merozoite invades an uninfected RBC and can be lost via two different routes. First, infected RBCs can die at a background rate μ. Second, infected RBCs burst to release merozoites after a period of α days (i.e., 1 day for the rodent malaria parasite, *P. chabaudi*). For simplicity, we omit immune responses that remove infected RBCs, although simulations of this model including a saturating immune response have delivered similar optimal conversion rate profiles (results not shown).

The dynamics of merozoites and gametocytes are described as

(3) $$\frac{dM}{dt}=\left(1-c(t)\right)\beta pR\left(t-\alpha \right)M\left(t-\alpha \right)S-pR\left(t\right)M\left(t\right)-\mu_{M}M\left(t\right)$$

(4) $$\frac{dG}{dt}=c\left(t\right)pR\left(t-\alpha \right)M\left(t-\alpha \right)S-\mu_{G}G\left(t\right)$$

where *c*(*t*) is the proportion of parasites in a given cohort of infected RBCs that become gametocytes after successful development (i.e., the conversion rate). We allow the conversion rate to vary over the course of infection, as has been observed in experimental data (Greischar et al., 2016b; Pollitt et al., 2011; Reece, Duncan, West, & Read, 2005). The burst size, β, is the number of merozoites released from each infected RBC surviving the developmental period. Merozoites die at a rate $\mu_{M}$, and gametocytes die at a rate $\mu_{G}$.

Equations (2)–(4) are defined for t>α. The dynamics of the initial inoculum of parasites, *I*_0_, are governed by

(5) $$\frac{dI}{dt}=pR\left(t\right)M\left(t\right)-\frac{I_{0}S}{\alpha }-\mu I$$

(6) $$\frac{dM}{dt}=\left(1-c\left(t\right)\right)\beta \frac{I_{0}S}{\alpha }-pR\left(t\right)M\left(t\right)-\mu_{M}M\left(t\right)$$

(7) $$\frac{dG}{dt}=c\left(t\right)\frac{I_{0}S}{\alpha }-\mu_{G}G\left(t\right)$$

(8) $$S=e^{-\mu t}$$

for t≤α.

### 2.2 Drug action

**Figure A.1:**
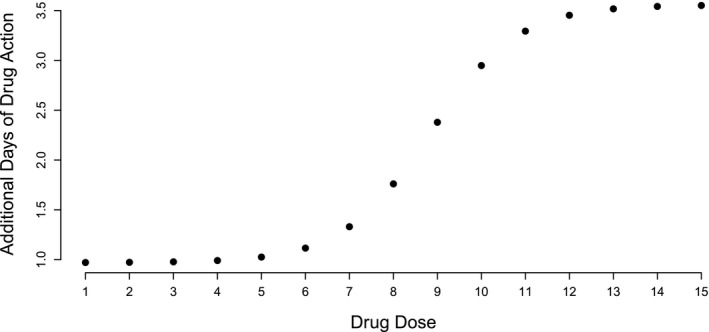
Drug dose affects duration of drug‐related parasite killing, but not the rate at which parasites are killed. Shown are the additional days of drug action, beyond the days when drugs are administered, when drugs are predicted to still be “active” (as defined in Huijben et al. 2013). Drug dose is expressed in mg/kg.

We incorporate the model of drug action presented in Huijben et al. (2013), which was parameterized to describe the consequences of pyrimethamine for *Plasmodium chabaudi* parasites (Landau, 1965) in infections of female C57BL6 mice (Schneider et al., 2012). According to this model, as long as the drug is present at a sufficiently high concentration in the host, it kills a fixed proportion (94%) of parasites each day. The underlying within‐host model assumed in Huijben et al. (2013) was in discrete‐time and cohorts of infected cells burst synchronously. To approximate this drug action in our model, we apply an additional death rate, $\mu_{d}$, to infected cells. By setting $\mu_{d}=-\ln\left(1-0.94\right)=2.81$, we ensure that ∼94% of infected cells die within the 1 day parasite developmental cycle. Different drug doses, *d*, modify the length of drug action, *L*, beyond the days the drug was administered (see Figure A.1 in Appendix A, for how L varies with dose):

(9) $$L=3.557-\frac{2.586}{1+e^{-8.821+d}}.$$

Therefore, parasites are subject to a drug‐induced mortality rate for each day that the drugs are administered, plus an additional *L* days afterwards. To explore the consequences of different strengths of drug treatment on optimal patterns of conversion rates, we simulate several treatment regimes: drug doses of 0–15 mg/kg, each administered for two consecutive days (days 11 and 12 postinfection). Determining the survival of infected RBCs (*S*) requires integrating these mortality rates over the delay α. For the case of drug‐treated infections, that survival term is given by

(10) $$S=\left\{\begin{array}{cc} \exp(-\mu t), & t<\alpha \\ \exp\left(-\left(\int_{t-\alpha }^{11}\mu d\omega +\int_{11}^{t}\mu +\mu_{d}d\omega \right)\right), & 11\leq t<\alpha +11,\\ \exp\left(-\left(\int_{t-\alpha }^{t}\mu +\mu_{d}d\omega \right)\right), & \alpha +11\leq t<L+12,\\ \exp\left(-\left(\int_{t-\alpha }^{12}\mu +\mu_{d}d\omega +\int_{12}^{t}\mu d\omega \right)\right), & L+12\leq t<L+12+\alpha ,\\ \exp(-\mu \alpha ), & \text{otherwise}. \end{array}\right.$$

**Figure A.2:**
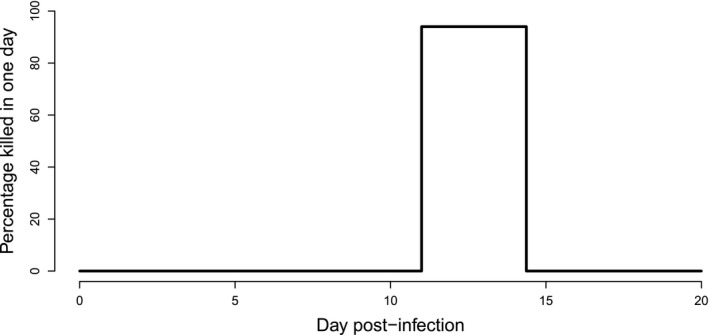
Schematic of drug action in our model, a stylized version of how pyrimethamine acts against *P.chabaudi*. In this example, drug treatment is composed of two doses of 9 mg/kg, administered on day 11 and 12. The last dose determines how long the drugs will persist in the host after treatment, here an additional ~2.4 days of drug action. Before and after drug action, drug‐related killing is zero.

Given our other model parameters, these treatment regimes encompass outcomes from a small, transient reductions in parasite loads, to a strong reduction in parasite load that would prevent further transmission on the timescale of our simulation. A schematic of the model of drug action is presented in Figure A.2 in Appendix A.

### 2.3 Optimization

**Figure A.3:**
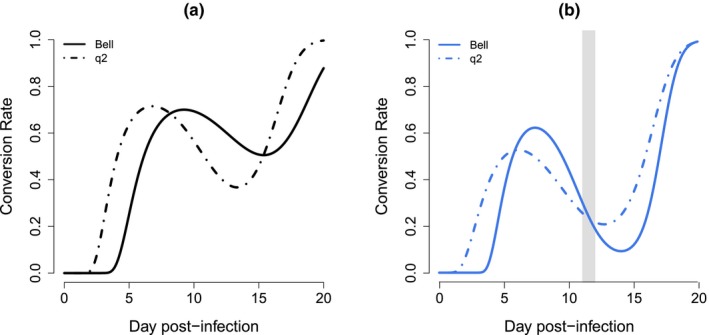
The optimal pattern of conversion over the course of infections, using equation “q2” in Huijben et al. (2006), rather than equation 11 of the main text to define fitness. (a) The black line shows the predicted best response in an untreated infection for the q2 fitness equation and the fitness equation proposed by Bell et al. (2010), used in this paper and marked “Bell”. (b) When infections are treated with a moderate drug dose (blue line, 8 mg/kg), parasites do better by reducing conversion, for both fitness functions. Drugs are administered on the days denoted by the grey bar.

To find optimal patterns of transmission investment, we use the optim function in R version 3.0.2 and define the cumulative transmission potential as our measure of fitness. This metric translates daily estimates of gametocyte density into the probability of that density resulting in an infected mosquito, assuming mosquitoes are abundant and biting hosts on a regular basis. The relationship between gametocyte densities and transmission probability is assumed to be sigmoidal, as has been experimentally derived for *P. chabaudi* by Bell et al. (2012). Using their parametrization, our fitness function is calculated as

(11) $$f\left(\eta \right)=\int_{0}^{\eta }\frac{e^{-12.69+3.6\;\log_{10}G\left(t\right)}}{1+e^{-12.69+3.6\;\log_{10}G\left(t\right)}}dt,$$

where *G*(*t*) is the gametocyte density at time point *t*, and η is the day postinfection at which our simulated infection ends. A sigmoidal relationship between gametocyte density and transmission success has also been reported for *P. falciparum* (Huijben et al., 2010) and gives similar results if used instead of the fitness function described here (see Figure A.3 in Appendix A). Our model describes early infection dynamics, before major adaptive immune responses develop. We therefore simulate a 20‐day infection over which we calculate the cumulative transmission potential, as has been done previously (Greischar et al., 2016a).

In a first set of optimizations, we define transmission investment to be a constant (c(t)=x, for all *t*) and determine the optimal time‐invariant conversion rate. Second, following Greischar et al. (2016a), we use cubic splines for the optimization of time‐varying conversion strategies, implemented in **R** with the “splines” package. Cubic splines require only four parameters to specify but allow considerable flexibility in the pattern of conversion over a 20‐day infection, and more complicated splines yield minimal fitness gains (Greischar et al., 2016a). Conversion rates must be constrained to vary between zero and one, so we take the complimentary log‐log of the value specified by the spline, that is, c(t)=exp(−exp(splinevalueattimet)). The starting values of the variables and the assumed value for each of the model parameters are given in Table 1, and each optimization is initiated by setting all spline parameters to an arbitrary starting guess of 0.5. Although no numerical optimization routine can guarantee finding a globally optimal solution, we sought to substantiate our findings by testing, for a given environment (i.e., drug dose), whether the putative optimal strategy for that environment outperformed the putative optimal strategies from other environments.

**Table 1:** Model parameters

Parameter	Description	Value or range	References
R*	Red blood cell density of a healthy mouse	8.5 × 10^6^ cells/μl	Savill, Chadwick, and Reece (2009)
λ	Maximal red blood cell production rate	3.7 × 10^5^ RBCs/μl	Savill et al. (2009)
μ	Red blood cell death rate	0.025/day	Miller, Råberg, Read, and Savill (2010)
*p*	Maximal per merozoite invasion rate	4 × 10^−6^/day	Mideo et al. (2008)
α	Bursting delay	1 day	Landau and Boulard (1978)
β	Burst size	10 merozoites	Mideo et al. (2008)
$\mu_{M}$	Merozoite death rate	48/day	Mideo et al. (2008)
$\mu_{G}$	Gametocyte death rate	4/day	Gautret, Miltgen, Gantier, Chabaud, and Landau (1996)
$\mu_{d}$	Drug‐induced death rate of infected cells	2.81/day	Adapted from Huijben et al. (2013)
*I*_0_	Initial dose of infected red blood cells	43.85965/μl	~10^4^ per mouse
*d*	Drug dose	0–15 mg/kg	

## 3 RESULTS

### 3.1 Constant conversion rates

**Figure 1:**
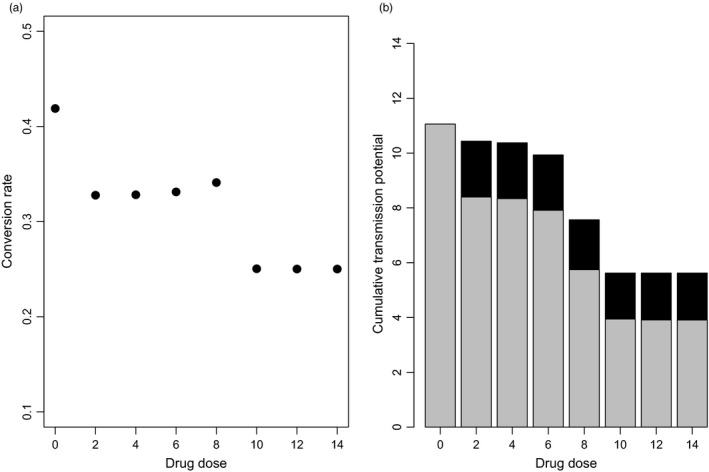
Lower conversion rates can buffer the effects of drugs. (a) Optimal constant conversion rates in the face of drug treatment (labelled as doses in mg/kg) are lower than in the absence of drugs. (b) As expected, drug treatment reduces parasite fitness (i.e., cumulative transmission potential). Grey bars indicate fitness when parasites are constrained to the drug‐free optimal conversion rate (~0.42). Black bars show the fitness gains achieved by adopting the dose‐specific optimal conversion rate (from A). With lower conversion rates, parasites are able to recoup some of the fitness that is lost due to drugs

Following previous work (Greischar et al., 2016a), we first constrained conversion rate in our within‐host model to be constant, and determined which single rate, maintained throughout the whole infection, produced the highest estimate of our parasite fitness proxy (i.e., cumulative transmission potential). In the absence of drugs, we find a similar optimal level of transmission investment as predicted previously (Greischar et al., 2016a). Drug treatment reduces the optimal level of transmission investment, with the lowest conversion rate predicted for the highest drug dose simulated (Figure 1a). We found little variation in the optimal transmission investment over low and moderate drug doses, as would be expected given our assumption that the drug dose changes the number of days of drug action rather than the killing rate (Huijben et al., 2013). For doses below 6 mg/kg, this formulation predicts little difference in the duration of drug action (see Figure A.1 in appendix A) or consequences for parasite fitness, as can be seen in Figure 1b. We therefore focus on 5 mg/kg, 8 mg/kg and 15 mg/kg as representative low, medium and high drug doses, respectively, for the remainder of our analyses. The step‐wise decrease in predicted conversion rates observed from a dose of 0 to 2 mg/kg and from a dose of 8 to 10 mg/kg closely follows the fitness effects that these increasing doses would have on parasites employing a non‐drug‐adapted conversion rate (Figure 1b, grey bars). Interestingly, we do not see a similar decrease in the predicted optimal conversion rate when the drug dose increases from 6 to 8 mg/kg, despite a substantial decrease in expected fitness for a non‐drug‐adapted strategy. An explanation for this may be found in the fact that a constant conversion rate represents a compromise, balancing the need to sustain a high enough asexual source population for conversion in the face of drug killing and having a sufficiently high conversion rate to successfully translate that asexual source population into onward transmission. Up to a dose of 8 mg/kg, slight increases in conversion rates can counteract lost fitness due to slight reductions in the asexual source population from higher doses. With a dose of 10 mg/kg or more, the asexual source population –and gametocytes– are reduced to such an extent that no more transmission is possible after the action of drugs. Therefore, the best option for a parasite is to restrain and increase the asexual source population that will be converted before the end of drug action.

We assume that all parasites within an infection are genetically identical; consequently, our fitness proxy is the cumulative probability of transmission over the course of infection. As our simulated infections run for 20 days, 20 represents the maximum cumulative transmission potential that would be achieved by a parasite genotype that sustained a sufficiently high gametocyte density to transmit to mosquitoes with 100% efficacy every day. Even in the absence of drugs, parasites cannot achieve 100% transmission efficacy at every point in the simulation, especially at the beginning of the infection when parasite numbers are low; hence, the maximum cumulative transmission potential is approximately 11 for the optimal level of fixed transmission investment of 0.42 in the absence of drugs (Figure 1b). The grey bars demonstrate the fitness achieved by parasites employing this same conversion rate (0.42) in the face of drug treatment. As expected, parasite fitness is lost as drug treatment reduces numbers. Some fitness can be recouped by adopting lower conversion rates (the drug dose‐specific optima, black bars). Indeed, with low drug doses, reduced conversion rates allow parasites to maintain roughly 90% of the fitness achieved in the absence of drugs.

### 3.2 Time‐varying conversion rates

**Figure 2:**
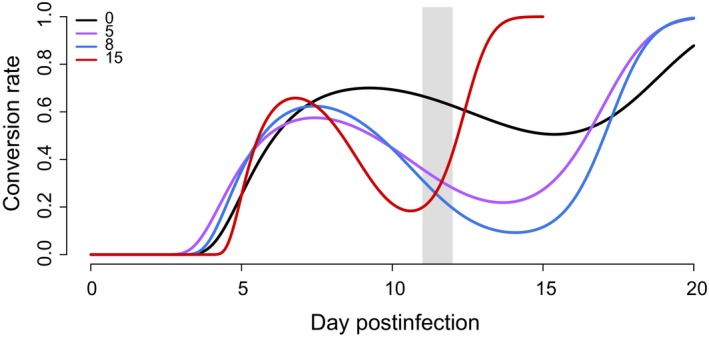
The optimal pattern of conversion over the course of infections. The black line shows the predicted best response in an untreated infection. When infections are treated (coloured lines), regardless of dose, parasites do better by reducing conversion (purple: low dose, 5 mg/kg; blue: medium dose, 8 mg/kg; red: high dose, 15 mg/kg). Drugs are administered on the days denoted by the grey bar. If drug treatment reduces the infection to a degree where parasites cannot expect any future transmission, then the best response for parasites is to terminally invest (as suggested by the red line). Note that the patterns diverge before drug treatment due to the constraints of our fitting regime; however, early differences in investment patterns contribute little to fitness differences (see text)

**Figure 3:**
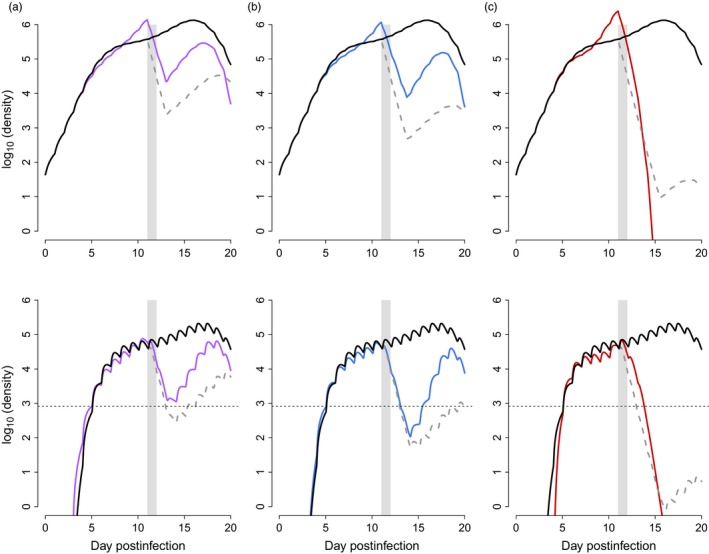
The within‐host dynamics of infected red blood cells (i.e., asexual parasites; top row) and gametocytes (bottom row). Coloured lines show dynamics when parasites are using the optimal conversion profiles for a given drug treatment (a: low dose, purple; b: medium dose, blue; c: high dose, red). The black lines show dynamics in the absence of treatment, for parasites using the optimal drug‐free pattern of conversion, while the dashed grey lines show how the different drug treatment regimes impact these dynamics if parasite life history patterns are unchanged from the drug‐free optimum. Grey bars denote the days of drug treatment and the horizontal lines in the bottom row indicate the gametocyte density at which there is a 10% probability of transmitting to a mosquito, according to Bell et al. (2012)

Next, we allowed the conversion rate to vary over the course of the infection and determined what pattern of transmission investment would maximize cumulative transmission potential (Equation (7)). The work of Greischar et al. (2016a) suggests that, in the absence of drug treatment, optimal patterns of conversion rate comprise roughly four distinguishable phases: (i) an “initial replication” phase where parasites delay gametocyte production to increase their numbers; (ii) a “peak conversion” phase where parasites dramatically increase transmission investment to capitalize on their large numbers; (iii) a “trough” where parasites reduce transmission investment to compensate for declining numbers in the face of resource limitation; and finally, (iv) “terminal investment, ” where parasites invest heavily into gametocyte production before the infection ends. We find qualitatively similar strategies (with the same four phases) in drug‐treated infections (Figure 2). The corresponding dynamics of infected red blood cells and gametocytes are shown in Figure 3. A key difference in the predicted optimal patterns of conversion in drug‐treated compared to untreated infections is an earlier and faster reduction in conversion rates (i.e., greater reproductive restraint) following the initial peak conversion (compare black to coloured lines in Figure 2). Comparing low and medium dose treatment regimes, we find that increasing dose is accompanied by greater reproductive restraint following treatment. The best response to a high drug dose is early terminal investment, which ultimately ends the infection (see infection dynamics in Figure 3c).

**Figure 4:**
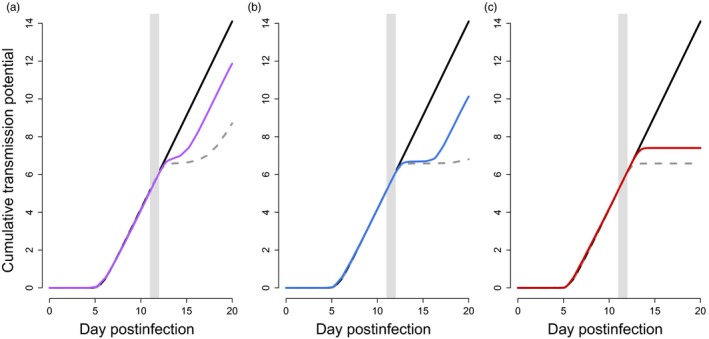
Cumulative transmission potential (fitness) over the course of infections. Given our fitness function, a parasite can maximally transmit with a probability of 1 each day, reaching a cumulative transmission potential of 20 at the end of the simulated infection. Black lines show the fitness obtained by a parasite adopting the drug‐free optimal pattern of conversion over the course of an untreated infection. Dashed grey lines show the consequences of drug treatment on parasites using that same strategy in the face of drug treatment: (a) low dose, 5 mg/kg; (b) medium dose, 8 mg/kg; (c) high dose, 15 mg/kg. Coloured lines show the fitness obtained by parasites using the drug dose‐specific optimal patterns of conversion (from Figure 2) in the face of drug treatment and indicate that parasites can recover some of the fitness lost due to drug treatment by altering patterns of conversion. Grey bars denote the days of drug treatment

**Figure 5:**
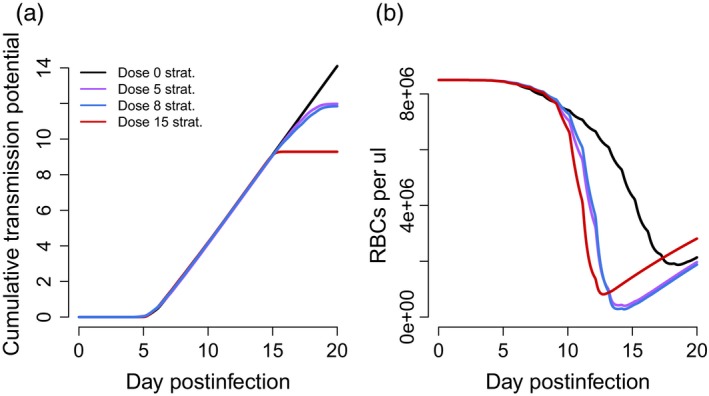
Consequences of parasite adaptation to drug‐treated infections. (a) The cumulative transmission potential in untreated infections where parasites employ different conversion rate strategies. Reproductive restraint in untreated infections produces only small transmission costs (purple and blue line) compared to strategies for untreated infections (black line), whereas terminating an infection early has bigger fitness consequences (red line). (b) The dynamics of uninfected red blood cells in those infections. Simulations assume optimal strategies for untreated infections (black), infections treated with a low dose (purple), medium dose (blue) and high dose (red). The reproductive restraint predicted for drug‐adapted strategies leads to earlier declines in RBCs and lower minimum values (i.e., greater anaemia) when infections are not drug‐treated

**Figure A.4:**
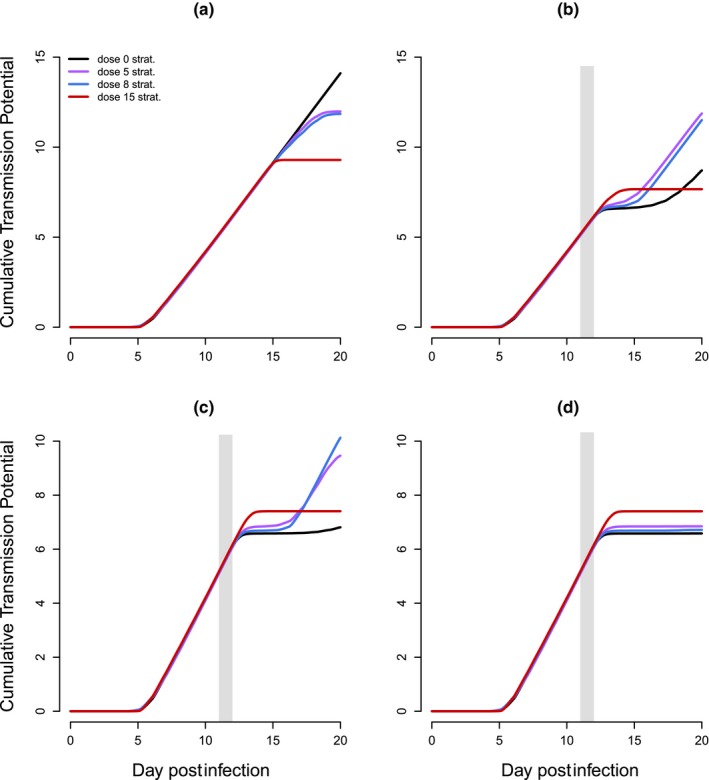
The cumulative transmission potential of different drug‐adapted strategies in untreated hosts (a), hosts treated with 5 mg/kg of drugs (b), 8 mg/kg (c), and 15 mg/kg (d). For each drug treatment, the putative optimal strategy against that dose outperforms the putative optimal strategies from other doses. Grey bars denote the days of drug treatment.

To identify the fitness consequences of these different strategies, we plot cumulative transmission potential over the course of infections. In Appendix A, we confirm that the putative optimal strategy against a given dose outperforms the putative optimal strategies from other doses (see Figure A.4). The optimal strategies—and the corresponding cumulative transmission potential—are similar prior to drug treatment (Figures 2 and 4, respectively). After drug treatment, the transmission investment strategies diverge, and there are clear costs to parasites that employ the incorrect strategy for the drug dose they encounter within the host (compare coloured to dashed grey curves in Figure 4). Specifically, in the absence of drug treatment, the optimal drug‐free strategy accrues fitness at nearly the maximal rate, corresponding to an almost 100% chance of transmitting to mosquitoes each day (black lines, Figure 4). But, this strategy performs successively worse in the face of increasing drug doses (dashed grey lines Figure 4; see also Figure 3 for corresponding infection dynamics). The optimal strategies for low, medium and high drug doses allow parasites to recoup a substantial portion of these fitness losses (coloured lines in Figure 4), attributable to greater reproductive restraint immediately after drug treatment (Figure 2). Notice that in the face of a high drug dose, the drug‐free strategy accrues no fitness following treatment (Figure 4c, dashed grey line), despite the fact that gametocytes are still circulating for days in those infections (Figure 3c, dashed grey line). This is because the densities are too low to achieve more than a negligible probability of transmission. In untreated infections, parasites that use reproductive restraint pay only a small fitness cost, whereas parasites employing strategies against high drug doses pay a more substantial fitness cost due to premature terminal investment (Figure 5a).

While reproductive restraint in response to treatment can, to some extent, buffer against the effects of drugs, our models predict that treatment still leads to reductions in parasite fitness and, importantly, reductions in transmission potential. As reproductive restraint necessarily means prioritization of asexual replication and it is these parasite stages that are most responsible for the virulence (harm) of a malaria infection, there may be consequences of shifting patterns of conversion at the host (or clinical) level. Drug treatment reduces infected RBC densities, even if parasites alter their conversion rates (Figure 3), but what if parasites employ drug‐adapted strategies in an infection that remains untreated? Figure 5b shows that, in an untreated host, infections composed of parasites using a drug‐adapted strategy (coloured lines) are predicted to result in much more rapid declines in uninfected RBC densities, and greater anaemia as measured by minimum RBC counts, compared to parasites using the best strategy in the absence of drugs (black line).

**Figure B.1:**
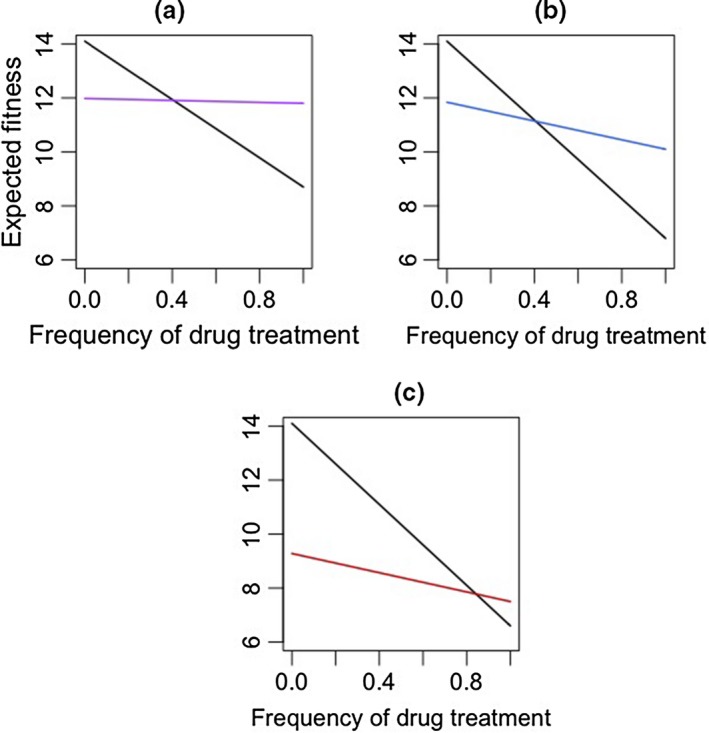
Expected fitness for different transmission investment strategies in a host population treated with a particular drug dose (A: low; B: medium; C: high) at a given frequency. Lines show the weighted average of fitness achieved in untreated and treated infections (i.e., solutions to Equations B.2). Black lines represent the transmission investment strategy predicted to be best in the absence of drug treatment (the “non‐drug adapted” strategy); coloured lines represent the transmission investment strategy predicted to be best in the face of a low drug dose (purple), medium drug dose (blue) or high drug dose (red).

Of course, the likelihood of a drug‐adapted strategy becoming fixed in the parasite population depends on the frequency that parasites encounter drug‐treated hosts, the benefits of altered patterns of conversion in a drug‐treated host, as well as the costs of that strategy in an untreated host. Using the fitness estimates for the different strategies in different environments (Table B.1 in Appendix B), we calculate the expected fitness for the drug‐adapted and non‐drug‐adapted strategies in a host population where some proportion of hosts are treated (Figure B.1). If *b* is the increase in fitness achieved by the drug‐adapted strain in the presence of drugs (i.e., the benefit), *c* is the reduced fitness of the drug‐adapted strain in an untreated host (i.e., the cost), and *f* is the proportion of infected hosts that are drug‐treated, then it is trivial to show (see Appendix B) that the drug‐adapted strategy has a higher fitness than the non‐drug‐adapted strategy when

(12) $$f>\frac{c}{c+b}.$$

Put another way, the drug‐adapted strategy will be favoured when the ratio of the benefits to costs of the strategy is greater than the relative frequency of encountering an untreated host:

(13) $$\frac{b}{c}>\frac{1-f}{f}.$$

Given our estimated fitnesses for the different strategies in different host environments, the drug‐adapted strategy will be favoured over the non‐drug‐adapted strategy when at least ~40% of infections are treated with a low or medium dose, or at least 86% of infections receive a high dose treatment. The early terminal investment strategy predicted to be optimal in the face of a high drug dose gains only a small fitness advantage in a treated host, while it suffers a large fitness cost in an untreated host (see also Table B.1), explaining why drug treatment would have to be very common to generate a sufficient selection pressure to favour that strategy.

## 4 DISCUSSION

The evolution of drug‐resistant parasites is a serious obstacle to the control of malaria (Dondorp et al., 2009; White, 2004). In addition to classical resistance mechanisms, we have shown that drug treatment can select for altered life history of malaria parasites and, specifically, changing patterns of allocation to transmission versus asexual parasite stages. Our work predicts that reproductive restraint is adaptive in drug‐treated infections, allowing parasites to partially compensate for the reductions in asexual densities caused by the drug. We also show that parasite adaptation to drug treatment could lead to worse outcomes for hosts that remain untreated, although as would be expected this outcome depends on the frequency with which parasites find themselves in treated hosts as well as the precise costs and benefits associated with different investment patterns in different environments.

Experimental evidence suggests that malaria parasites do alter their investment in transmission in response to drugs. Reece, Ali, Schneider, and Babiker (2010), for example, found a decrease in conversion in human malaria parasites exposed to low doses of drugs in vitro, as our model predicts, unless they were known to be “classically” drug‐resistant parasites, which showed no change in investment (a result that highlights the multiple routes available for mitigating the effects of drugs). A similar study found no effect of drug dose on conversion rates (Peatey et al., 2009), and an in vivo rodent malaria experiment suggested that subcurative drug doses lead to increased conversion (Buckling, Taylor, Carlton, & Read, 1997). In contrast to the results of Reece et al. (2010), these latter two examples show parasite responses that appear maladaptive in the light of our model results, raising at least two further questions. First, have parasite strategies been accurately measured? Inferring conversion rates is fraught with difficulties that have only recently been resolved (Greischar et al., 2016b), and reanalysis of past data sets could reconcile the discrepancy between theoretical predictions and empirical estimates of transmission investment. Second, are parasites capable of evolving adaptive transmission strategies to the novel selection pressure of drug treatment? Addressing this question means evaluating whether the parasites in these experiments would have achieved greater fitness than ones with different responses, which necessitates tools for manipulating parasite strategies. Advances in understanding the molecular pathways associated with commitment to gametocytogenesis (e.g., Brancucci, Goldowitz, Buchholz, Werling, & Marti, 2015) may bring such tools for experimental manipulation into reach.

Recent work has focused on dormancy as another nonclassical resistance mechanism thought to be employed by malaria parasites (e.g., Codd, Teuscher, Kyle, Cheng, & Gatton, 2011; Hott et al., 2015; Paloque, Ramadani, Mercereau‐Puijalon, Augereau, & Benoit‐Vical, 2016; Teuscher et al., 2010). This delayed development confers protection against the effects of fast‐acting drugs that decay rapidly within a host, but whether such a strategy would be beneficial against drugs with longer half‐lives is unclear. Parasites can stall their intra‐erythrocytic development for many days, but only a small fraction—less than two per cent—appear to successfully recover and resume development even at low drug doses (Teuscher et al., 2010). It is not clear that such a low percentage of parasites entering dormancy can explain malaria dynamics in patients (Saralamba et al., 2011). Further, the fitness consequences of dormancy are not intuitive: surviving the effects of drugs is clearly good from the parasite's perspective, but stalling development means stalling production of transmission stages and missing out on any transmission opportunities during the dormant phase. In contrast, parasites can recover substantially more than two per cent of their numbers by modifying transmission investment under some treatment regimes. Indeed, Figure 3 suggests that parasite densities can actually increase by an order of magnitude or more within less than 4 days and this modified life history translates to fitness gains (Figure 4). It is interesting to consider how these two mechanisms of nonclassical resistance would affect host health. At least in the short term, dormancy should reduce pathology associated with parasite replication as well as immunopathology, while reduced investment in transmission is likely to do the opposite.

We have shown that, in principle, altered life history can protect against the effects of drugs, and while we have used a model of drug action that was parameterized for a particular drug (pyrimethamine; Huijben et al., 2013), the phenomenological description we employ should capture the effects of many different drugs. Although there will be differences among individual hosts in drug metabolism that would affect, for example, the duration of drug action, our exploration of a range of drug doses should capture much of this variation. One exception to this generality is drugs that directly target gametocytes (e.g., primaquine, White, Ashley, et al., 2014). The relative susceptibility of asexuals and gametocytes to the drug will alter the costs and benefits of producing each stage, so different drugs may be expected to have different effects on optimal patterns of transmission investment. For example, a drug with a strong gametocidal effect may generate an advantage to reproductive restraint when drugs are present but promote the production of surplus gametocytes to compensate for those killed by drugs when drugs have cleared or may promote earlier production of gametocytes to compensate for lost transmission opportunities during drug treatment. Predicting evolutionary trajectories in response to such drugs will require precise calibration of the relative susceptibility of different parasite stages.

We have also ignored within‐host competition and thus evolution operating at the within‐host scale, but where malaria is endemic, multigenotype infections are the rule rather than the exception (e.g. Baruah, Lourembam, Sawian, Baruah, & Goswami, 2009; Juliano et al., 2010). Previous theoretical and experimental work shows that competition favours reproductive restraint (Greischar et al., 2016a; Greischar, Reece, et al., 2016; McKenzie & Bossert, 1998; Mideo & Day, 2008; Pollitt et al., 2011), so it is possible that our prediction of that same response in the face of drug treatment would remain unchanged. However, just as there is genetic variation for competitive ability (Bell, De Roode, Sim, Read, & Koella, 2006; de Roode, Helinski, Anwar, & Read, 2005; de Roode et al., 2005), there is likely to be genetic variation in sensitivity to drugs (and in *P. falciparum*, differentially sensitive genotypes may often share a host; e.g., Mideo et al. (2016)). If variation in drug sensitivity is unrelated to transmission investment, then it would alter the costs and benefits to different parasite genotypes of altering that investment. Modelling the dynamic consequences of competition and the interplay between different sources of resistance on the evolution of parasite life history would be an interesting route for future investigation. Importantly, there may also be genetic variation in the shape of the relationship between within‐host gametocyte densities and the probability of transmission to mosquitos. As far as we are aware, this relationship has been quantified only a few times and only for a few distinct strains (Bell et al., 2012; Huijben et al., 2010; Paul, Bonnet, Boudin, Tchuinkam, & Robert, 2007). While the qualitative shapes of these relationships remain the same, there are quantitative differences in their parametrization. We found that these differences did not alter our predictions (see Figure A.3 in Appendix A), but further empirical exploration of this relationship is warranted, as is theoretical investigation of how any quantitative changes in this relationship alter evolutionary predictions.

While our model allows for variation across infections treated with different drug regimes and variation over time within infections, our heuristic analysis also constrains variation at both of these scales. First, to determine when evolution should favour a drug‐adapted strategy, we assumed that there were only two strategies available to parasites: the pattern of transmission investment predicted to be best in an untreated host or the one predicted to be best in the presence of a particular drug dose. In a heterogeneous host population, some intermediate parasite investment strategy may perform better than either of these two “extremes”. Second, our model does not allow for parasites to directly receive and respond to cues within infections; that is, it is not a model of plasticity. Put another way, the model implicitly assumes that parasites have perfect knowledge about the timing of drug treatment (which does not vary across treated hosts) and optimal patterns of investment may allow parasites to, in effect, prepare in advance for drug treatment. This scenario may not be too far from reality in some areas. Drug doses are standardized by WHO guidelines (WHO 2015b), and hosts likely seek treatment when symptoms appear, which generally correlates with peak parasite density (Kachur et al., 2006), although there will be variation across individual hosts in the timing of early dynamics. How much fitness could be gained by allowing parasites in our model to detect and respond to drug treatment more directly is unclear, as our results suggest that differences in investment early in infections (and, in particular, before drug treatment) have little effect on parasite fitness. Consistent with this, Greischar et al. (2016a) found that investing little in transmission at the beginning of infections is adaptive in untreated hosts, regardless of other changes to the within‐host environment. Thus, it seems unlikely that allowing parasites more flexibility in pretreatment patterns of investment would result in different life history strategies than we have predicted. On the other hand, if parasites could respond plastically to the presence of drugs in the within‐host environment (instead of through evolutionary change, as we have focused on), then this would avoid the negative consequences for host health we report.

The evolution of classical resistance is the expected result of using chemical interventions to kill parasites (or, in evolutionary terms, reduce their fitness), but, as we have shown, failing to consider the potential for nonclassical resistance, like life history evolution, can yield overly optimistic predictions about the epidemiological or clinical effects of those interventions. Similarly, Lynch, Grimm, and Read (2008) used models to investigate the influence of different antihelminth interventions on nematode life history, finding that disease control programs may frequently select for increasingly fecund worms, with ramifications for clinical outcomes and onward transmission. In an experimental system, filarial nematodes altered their reproductive schedules in the presence of specialized immune cells, producing transmissible stages faster and in greater numbers (Babayan, Read, Lawrence, Bain, & Allen, 2010). As these are the same immune cells on which current experimental vaccines rely, this work suggests that nematodes could reduce the benefits of vaccination through plasticity in life history. Further, the mosquitoes that transmit malaria and other diseases can also respond to intervention efforts with nonclassical resistance, including, for example, changes in feeding behaviour or timing to avoid insecticide‐treated bednets (Gatton et al., 2013; Sokhna, Ndiath, & Rogier, 2013).

An important question is how treatment recommendations would change in the light of our predictions about optimal malaria parasite life histories. Regardless of the life history shifts we predict here, parasite fitness and within‐host densities are reduced by drug treatment. This suggests that despite the evolution of nonclassical resistance, drug treatment offers epidemiological and clinical benefits. Those benefits are not as great as they would be in the absence of life history evolution and, importantly, any hosts that remained untreated could be worse off if drug‐adapted strategies became fixed in the parasite population. Further, as a result of altered patterns of transmission investment, parasites could maintain higher within‐host densities in the face of drug treatment, potentially facilitating the evolution of classical resistance. The theory developed here provides a basis for assessing the constraints and limits on parasite life history evolution in response to human interventions.

## Appendix A Supplementary figures

### A.1

Huijben et al. (2013) parameterised a model for the action of pyrimethamine against *Plasmodium chabaudi* in mice, finding that the dose of drugs affected the duration of drug action. We show this relationship (i.e., solutions to Equation (9) of the main text) in Figure A.1. A schematic of the full drug action model is presented in Figure A.2. In Figure A.3, we explore the effects of using a different fitness function on the predicted optimal patterns of investment in the absence of drug treatment and with a medium dose drug treatment. Finally, Figure A.4 shows the fitnesses achieved by different strategies in different environments (i.e., untreated or treated hosts). In each case, the optimal strategy predicted for a given environment outperforms the predicted optimal strategies for other environments.

## Appendix B Fitness calculations

### B.1

Imagine the following set of fitnesses for a non‐drug‐adapted and a drug‐adapted pattern of transmission investment (first subscript 0 or D, respectively) of malaria parasites in untreated and treated host (second subscript 0 or D, respectively).

(B.1) $$\begin{array}{c} w_{0,0}=a\\ w_{0,D}=a-d\\ w_{D,0}=a-c\\ w_{D,D}=a-d+b \end{array}$$

where d is the reduction in fitness of the non‐drug‐adapted strain due to drug treatment (i.e., the drug effect), c is the reduced fitness of the drug‐adapted strain in an untreated host (i.e., cost to “resistance”), and b is the increase in fitness achieved by the drug‐adapted strain in the presence of drugs (i.e., the benefit of “resistance”).

We can write the expected fitness of the two different strategies in a host population, where a proportion, f, of hosts receive drug treatment:

(B.2) $$\begin{array}{c} E\left[w_{0}\right]=fw_{0,D}+\left(1-f\right)w_{0,0}\\ E\left[w_{D}\right]=fw_{0,D}+\left(1-f\right)w_{D,0} \end{array}$$

Substituting the fitness expressions from B.1 into B.2 and rearranging, we find that the drug‐adapted strategy has a higher fitness when

(B.3) $$f>\frac{c}{c+b}$$

Put another way, the drug‐adapted strategy will be favoured when the ratio of the benefits to costs of the strategy is greater than the relative frequency of encountering an untreated host:

(B.4) $$\frac{b}{c}>\frac{1-f}{f}$$

In Table B.1 we list the cumulative transmission potential (as predicted by our model), over a 20‐day simulated infection, for each of the predicted drug‐adapted strategies, in the presence and absence of drug treatment, as well as the non‐drug‐adapted strategy in each of these environments. From these values we can plot the expected fitness of different strategies (i.e., solutions to Equations B.2) over different values of f (Figure B.1). We see that over a range of f values, the non‐drug adapted strategies performs better on average than the drug adapted strategy, for all drug doses, but above a given f value, the drug‐adapted strategy will be favoured. From the fitness values, we can also calculate b and c for each of the drug‐adapted strategies (Table B.2). Plugging these costs and benefits into Equation B.3, gives rise to the frequencies of drug treatment required to favour the drug‐adapted over the non‐drug adapted strategies reported in the main text (i.e., the intersection of the lines in Figure B.1).

**Table B1:** Estimated fitness values (i.e., cumulative transmission potential) for different transmission investment strategies in different host environments, as predicted by the model presented in the main text

Strategy	Environment (drug dose)			
	0	5	8	15
0	14.1	8.7	6.8	6.6
5	11.98	11.8		
8	11.84		10.1	
15	9.28			7.4

**Table B2:** Calculated benefits, b, and costs, c, of drug‐adapted strategies

Strategy	Effects of ‘resistance’	
	b	c
5	3.1	2.12
8	3.3	2.26
15	0.8	4.82
